# Unique metabolites protect earthworms against plant polyphenols

**DOI:** 10.1038/ncomms8869

**Published:** 2015-08-04

**Authors:** Manuel Liebeke, Nicole Strittmatter, Sarah Fearn, A. John Morgan, Peter Kille, Jens Fuchser, David Wallis, Vitalii Palchykov, Jeremy Robertson, Elma Lahive, David J. Spurgeon, David McPhail, Zoltán Takáts, Jacob G. Bundy

**Affiliations:** 1 Department of Surgery and Cancer, Imperial College London, Sir Alexander Fleming Building, London SW7 2AZ, UK; 2 Department of Symbiosis, Max-Planck-Institute for Marine Microbiology, Bremen 28359, Germany; 3 Department of Materials, Imperial College London, London SW7 2AZ, UK; 4 Cardiff School of Biosciences, Cardiff University, Park Place, Cardiff CF10 3AX, UK; 5 Bruker Daltonik GmbH, 28359 Bremen, Germany; 6 Department of Chemistry, Chemistry Research Laboratory, University of Oxford, Mansfield Road, Oxford OX1 3TA, UK; 7 Centre for Ecology and Hydrology, Maclean Building, Benson Lane, Crowmarsh Gifford, Wallingford, Oxon OX10 8BB, UK

## Abstract

All higher plants produce polyphenols, for defence against above-ground herbivory. These polyphenols also influence the soil micro- and macro-fauna that break down plant leaf litter. Polyphenols therefore indirectly affect the fluxes of soil nutrients and, ultimately, carbon turnover and ecosystem functioning in soils. It is unknown how earthworms, the major component of animal biomass in many soils, cope with high-polyphenol diets. Here, we show that earthworms possess a class of unique surface-active metabolites in their gut, which we term ‘drilodefensins'. These compounds counteract the inhibitory effects of polyphenols on earthworm gut enzymes, and high-polyphenol diets increase drilodefensin concentrations in both laboratory and field populations. This shows that drilodefensins protect earthworms from the harmful effects of ingested polyphenols. We have identified the key mechanism for adaptation to a dietary challenge in an animal group that has a major role in organic matter recycling in soils worldwide.

## Abstract (web summary)

Little is known about how detritivorous invertebrates cope with high levels of defensive plant polyphenols. Here, Liebeke *et al*. identify a new class of surface-active metabolites in earthworms exposed to high-polyphenol diets, and show that they play a protective role against precipitation of proteins.

## Graphical abstract

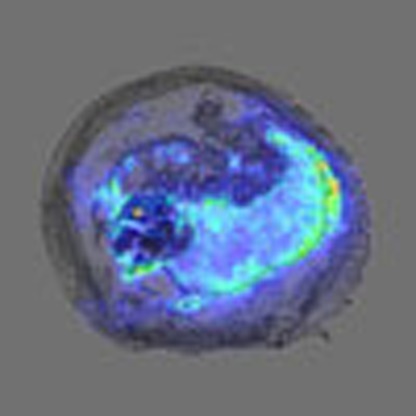

An estimated 3.5 × 10^10^ tonnes of terrestrial leaf litter are turned over worldwide each year in soils^1^. Plants produce polyphenols as a defence against above-ground herbivory^2^, but polyphenols can also have indirect effects on the soil micro- and macrofauna that break down plant leaf litter^3^,^4^. Polyphenols are found ubiquitously in plants, and often at high concentrations (for example, between 1 and 25% leaf dry weight^5^), and so the rate of transfer of soluble polyphenols alone into soil could reach levels of >200 kg ha^−1^ per year, from a combination of throughfall and leaching from decomposing leaf litter^6^. They are a complex group of chemicals with varying degrees of polymerization, ranging from simple phenols, phenolic acids and flavonoids to oligomers and polymers with tannins and lignin as main groups^7^. Polyphenolic compounds bind to and precipitate soluble proteins^8^,^9^ which can inhibit enzyme activity^10^. Mammals counteract these deleterious effects by producing proline-rich salivary peptides which sequester polyphenols into insoluble complexes^11^. In contrast, some invertebrates produce gut surfactants in response to a polyphenol-rich diet^12^,^13^,^14^,^15^, but little is known about their structure in general and if they are directly involved in the interaction with polyphenols. Earthworms form the major component of animal biomass in many soils, and are considered ecosystem engineers owing to their key role in organic matter turnover^16^,^17^. Earthworms prefer low-polyphenol-content plant material if available^18^, indicating that polyphenols can have a negative effect on them, but so far it is not known how earthworms cope biochemically with high-polyphenol diets as found in many habitats.

Here, we show that a number of different dialkylfuransulfonate metabolites are found in all earthworm species that we have tested so far, but appear to be restricted to earthworms. These compounds were present in the anterior part of the earthworm gut, implying they might act as gut surfactants. We established that the major metabolite (2-hexyl-5-ethyl-furan-3-sulfonate) has similar surface activity to commercial products such as SDS, and reduces soluble protein precipitation by polyphenols without affecting the activity of earthworm gut enzymes. Furthermore, exposing earthworms (both laboratory and field populations) to high-polyphenol diets increased their surfactant metabolite concentration. Together, our results suggest that these metabolites, which we term here ‘drilodefensins', play a role in protecting earthworms from the high levels of polyphenols in their diet and thus help earthworms to live in diverse habitats.

## Results

### Furansulfonic acid metabolites are localized to the gut

**Figure 1:**
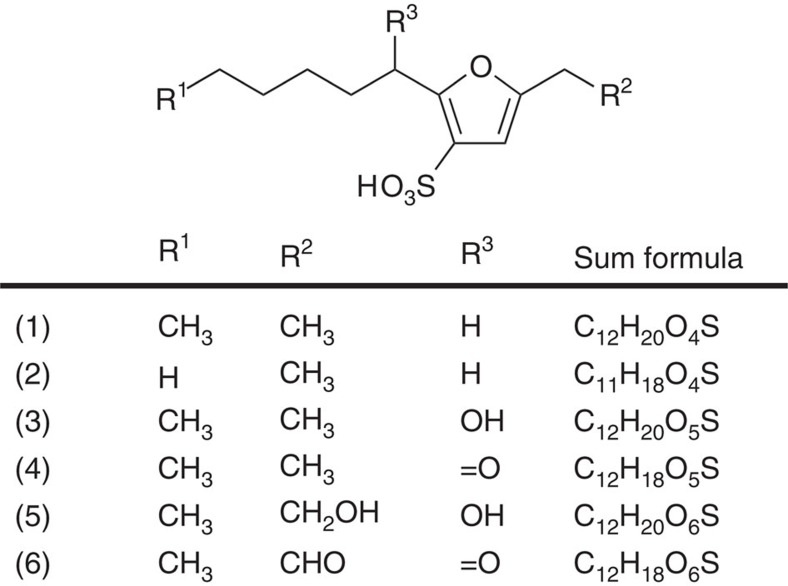
Structures of earthworm 2,5-dialkylfuran-3-sulfonic acid metabolites 1–6 NB that compounds **3**–**6** are based on mass spectrometry data only, and do not yet have associated NMR data.

We used untargetted metabolic profiling (metabolomics) to search for potential surface-active compounds (Supplementary Table 1) in the gut fluid of earthworms (obtained either by dissection or by squeezing out the gut contents). We detected only common primary metabolites without obvious surface-active structural properties, that is, we did not observe any metabolites similar to other small molecule gut surfactants from different species (Supplementary Fig. 1). However, earthworm samples can be labile because of unusually high residual enzyme activity^19^, potentially requiring techniques for *in situ* detection, and so we used a combination of complementary imaging mass spectrometry (IMS) techniques to explore metabolite distributions in snap-frozen and cryosectioned earthworms. IMS is opening up many areas of life sciences investigation, by adding critical information about tissue and cellular localization to biochemistry^20^,^21^,^22^. We first used high mass resolution IMS (MALDI-MS (matrix-assisted laser desorption/ionization) and DESI-MS (desorption electrospray ionization)) to identify which organ(s) or tissue(s) contained potential biosurfactant molecules. We were particularly interested in abundant compounds, as metabolites that play a direct functional role on the basis of their chemical properties (as opposed to signalling molecules, for instance) will tend to be present in high concentration. The highly abundant ion 259.1013 Da was localized to the earthworm gut (and particularly the gut wall region) in foregut sections, but not in tail sections (Supplementary Fig. 2a). This *m*/*z* value is consistent with a molecular formula of C_12_H_19_O_4_S^−^ (calc. 259.1010 Da), which most likely represents the [M-H]^−^ ion of the earthworm metabolite with proposed structure 2-hexyl-5-ethyl-furan-3-sulfonate (compound **1**; Fig. 1). This compound has been previously described as a metabolite in earthworms, but with no functional role assigned^23^. We verified the structure by synthesizing an authentic standard, and comparing the spectroscopic data (Supplementary Fig. 3). A related set of previously undescribed furan sulfonate metabolites (compounds **2**–**6**; Fig. 1) were also co-localized to the gut (Supplementary Fig. 4). The presence of these compounds in earthworms was confirmed by additional Ultra-Performance Liquid Chromatography (UPLC)-MS experiments of tissue extracts, giving unique retention times and mass spectra (Supplementary Fig. 5). The proposed structures for (**2**–**6**) are based on mass spectrometry fragmentation (MS^n^) experiments, either via direct infusion MS^n^ or on-tissue desorption electrospray ionization (ESI)-MS^n^ (Supplementary Fig. 6; Supplementary Table 2). Compound **2** was also further confirmed by synthesis of an authentic standard; compounds **3**–**6** remain putative structures as they do not yet have associated NMR data. The identified compounds are strong acids with a lipophilic alkyl chain, so are likely to be surface active.

**Figure 2:**
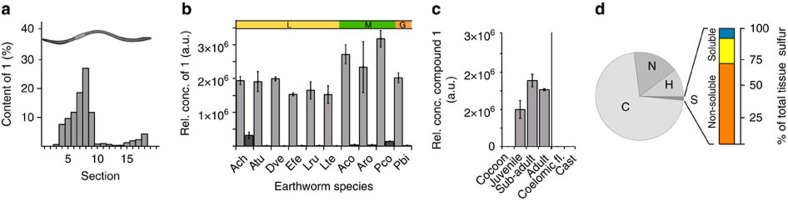
Drilodefensins are ubiquitous and abundant earthworm metabolites. (**a**) Quantitative longitudinal distribution of **1** in sections of an earthworm. (**b**) Abundance of **1** and **2** in earthworm species from three different families, yellow—Lumbricidae (L); green—Megascolecidae (M); orange—Glossoscolecidae (G); (**c**) Relative abundance of **1** during different earthworm developmental states and in earthworm exudates. The average±s.d., *n*=5 is shown for data in **b** and **c**. (**d**) Elemental composition of *L. rubellus* tissue; the bar chart represents sulfur content in non-extractable (∼70%, orange) and extractable (soluble) fractions, with the soluble fraction further separated into drilodefensins (20%, yellow) and other small molecules (10%, blue). Ach, *Allolobophora chlorotica*; Atu, *Aporrectodea tuberculata*; Dve, *Dendrobaena veneta*; Efe, *Eisenia fetida*; Lru, *Lumbricus rubellus*; Lte, *Lumbricus terrestris*; Aco, *Amynthas cortices*; Aro, *Amynthas rodericensis*; Pco, *Pontoscolex corethrurus*; Pbi, *Pithemera bicincta.*

**Figure 3:**
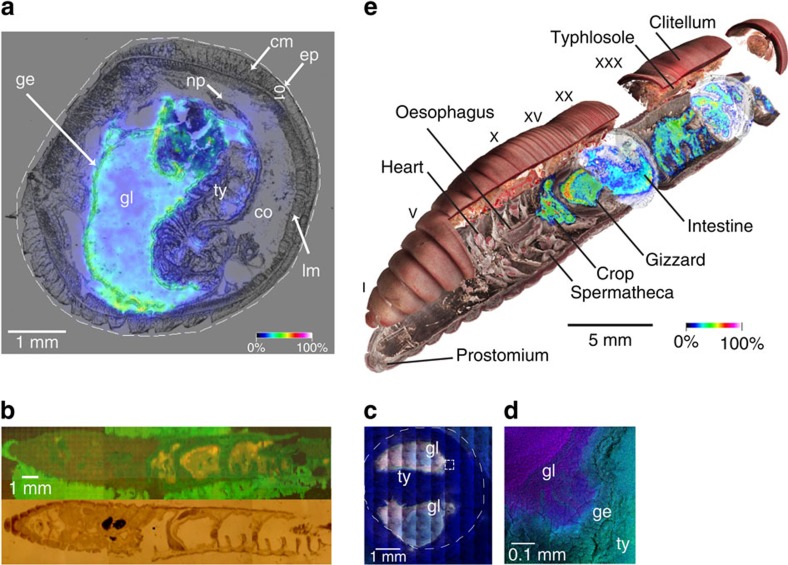
Drilodefensins are found solely in the earthworm gut. Transverse tissue cryosections were analysed by high-resolution mass spectrometry imaging. (**a**) MALDI-FTICR-MS, compound **1** (*m*/*z* 259.1004±0.001 Da M–H^+^) distribution overlaid on optical image from tissue transverse section in the foregut region; (**b**) TOF-SIMS analysis of a longitudinal cross-section through middle plane of earthworm, compound **1** in orange (*m*/*z* 259 Da), upper image; optical light-microscopic image, lower image. (**c**) TOF-SIMS derived ion-map for compound **1** in a full cross-section near the clitellum region, right image (**d**) shows higher spatial resolution image from white inset in (**c,** left image) showing **1** (*m*/*z* 259 Da) in purple and phosphocholine (*m*/*z* 184 Da) derived from tissue material in green. (**e**) Drilodefensin (compound **1**) distribution in an earthworm, schematic multi-modal three-dimensional model based on micro-computed tomography integrated with IMS data. The colour scale in **a** and **e** represents the relative abundance of *m*/*z* 259.1004. Labelling: ep, epithelium; cm, circular muscle; lm, longitudinal muscle; ge, gut (intestinal) epithelium; gl, gut lumen; np, nephridial tubule profiles (in coelomic cavity); ty, typhlosole fold.

Compound **1** was not evenly distributed along the alimentary system of the worm (Fig. 2a), with the highest concentrations in the foregut region. In comparison, other high-abundance metabolites like amino acids were more evenly distributed along the worm body (Supplementary Fig. 2b). In order to prove that compound **1** is located directly in the intestinal organs we used high spatial resolution IMS (time-of-flight secondary ion mass spectrometry, TOF-SIMS), which showed a clear localization of compound **1** next to the gut epithelium and within the gut lumen (Fig. 3a,c; Supplementary Fig. 7) and not in adjacent tissue. In addition, based on a coronal section, compound **1** was present in all organs of the alimentary system apart from the oesophagus and mouth (Fig. 3b). This is consistent with our hypothesis, as a biosurfactant defence compound would need to be present at an early point of contact between polyphenols and soluble proteins within the digestive system, analogous to the occurrence of proline-rich defensive peptides in vertebrate saliva^11^.

### The metabolites are unique to earthworms

These dialkylfuransulfonates appear to be unique to earthworms, as they are not found in their closest relatives, for example other clitellate annelids such as enchytraeids, leeches or naidids (Supplementary Fig. 8). There are high levels of compound **1** and moderate levels of **2** in all earthworm species that we have studied to date (14 species, including members from three different families: Lumbricidae, Glossoscolecidae and Megascolecidae; Fig. 2b), and **1** has also been reported in a non-lumbricid Australian earthworm^24^. During the different life stages of earthworms an increased amount of compound **1** was observed (Fig. 2c), with cocoons being free of compound **1**.

Unlike other sulfur-containing secondary metabolites in animals, which are often only found at low abundance, the dialkylfuransulfonates are present in high concentration in earthworms, and so potentially play a major role in the earthworms' sulfur budget. We determined the total tissue sulfur content of earthworms (7.2–8.0 mg g^−1^ dry weight), as well as the sulfur content of different subcellular fractions. Compound **1** comprises about 20% of total earthworm sulfur, and 70% of the sulfur from small molecules (Fig. 2d), such that earthworm sulfur biochemistry must have undergone substantial adaptation to meet this demand. Furthermore, compound **1** comprises ∼1.3% of total earthworm biomass dry weight (based on sulfur determination).

### The metabolites act as biological surfactants

**Figure 4:**
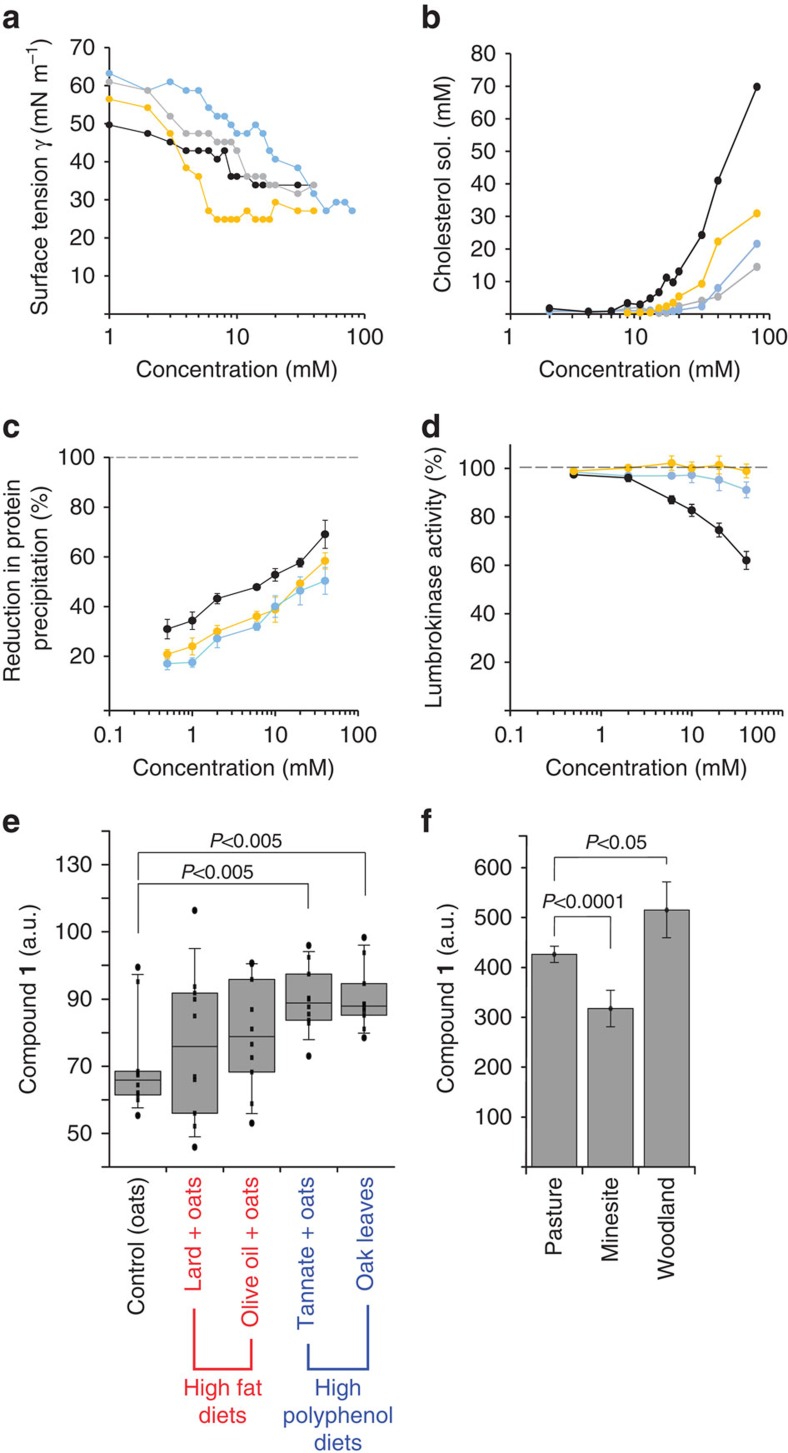
Gut surfactant levels are influenced by dietary polyphenols, and protect proteins from polyphenol precipitation while retaining earthworm gut enzymatic function. Influence of different concentrations of compound **1** (yellow)**, 2** (blue), cholic acid (grey) or synthetic surfactant SDS (black) on (**a**) surface tension in buffered solution, (**b**) cholesterol solubility, (**c**) precipitation of protein (BSA) by tannin and (**d**) enzymatic activity of an earthworm gut protease (lumbrokinase), experiments were replicated three times. The levels of compound **1** change in response to (**e**) diets containing different levels of either lipid or polyphenols (2 weeks exposure, 10 earthworms per treatment, shown is the mean (±95% confidence interval)), and (**f**) different leaf litter types of natural soils (lifetime exposure, specimens collected from field populations, number of specimens: pasture *n*=240, mine sites *n*=37, woodland *n*=21), error bars are s.d.; *P* values, Student's *t*-test.

To assess the surfactant properties of these compounds, we tested synthetic pure standards of the most abundant compounds **1** and **2** *in vitro*. Compound **1** dose responsively decreased the surface tension of aqueous buffer and started to form micelles at 6 mM (critical micellar concentration), which is comparable to other biological and synthetic surfactants (for example the bile acid cholic acid, 11 mM; and the synthetic compound SDS, 8 mM; Fig. 4a). Compound **2** was less surface active with a critical micellar concentration of 30 mM. The dialkylfuransulfonates are clearly biological surfactants; however, gut surfactants could have two possible biological functions: they could protect against phenols, and/or could act as functional analogues of bile acids (which are not found in earthworms) by solubilizing lipids. Both possibilities were tested separately. Firstly, the ability of compounds **1** and **2** to solubilize cholesterol (an essential dietary nutrient for earthworms^25^) was tested: compound **1** was more effective than the mammalian metabolite cholic acid at solubilizing cholesterol, although not as powerful as SDS (Fig. 4b). Secondly, though, compound **1** and **2** also reduced the precipitation of soluble proteins by polyphenols *in vitro*, without affecting the activity of an earthworm gut enzyme (lumbrokinase). Synthetic surfactants reduced protein precipitation to a similar extent, but at the cost of reducing enzyme activity (Fig. 4c,d).

### The surfactants are protective against dietary polyphenols

These experiments did not clearly distinguish between lipid solubilization or polyphenol defence as potential functions of these metabolites, and so earthworms were kept for 2 weeks in soil microcosms with either high-fat or high-polyphenol diets in order to determine the *in vivo* response. The food sources were comprised of one control diet (oats only), two with increased lipid content (amended with either olive oil or animal fat) and two with high polyphenol levels (amended with either a pure standard of tannic acid, a typical polyphenol compound, or with oak leaves, representing an ecologically relevant polyphenol-rich natural food for earthworms). High-fat diets increased compound **1** levels, but not significantly, whereas both the high-polyphenol diets led to an increase of ∼50% (*P*<0.01, Student's *t*-test, *n*=10 each treatment) in compound **1** (Fig. 4e). Consistent with these results from manipulative laboratory experiments, autochthonous earthworms from woodland sites (that is, exposed to greater amounts of high-polyphenol leaf litter) had higher levels of compound **1** compared with earthworms from grassland sites, which in turn had higher levels than earthworms from mine sites with very low levels of vegetation cover (Fig. 4f).

## Discussion

The combination of the intestinal localization, surface activity, protective effect against precipitation of proteins by polyphenols, and increase in concentration in response to polyphenols in both laboratory and wild populations, indicates that dialkylfuransulfonates represent a biochemical adaptation of earthworms that helps them to cope with polyphenol-rich plant diets. We propose naming these metabolites *drilodefensins* (from *megadrile*, the earthworm group of terrestrial clitellate annelids). Given that earthworms allocate so many resources to drilodefensins (making up 1% or more of the total dry biomass, and 20% of the total sulfur budget) they are clearly critically important to earthworms. Other terrestrial detritivores are also therefore likely to possess biochemical adaptations such as gut surfactants as defences against polyphenols^14^,^15^, although there is no reason to believe that they would be chemically related to the drilodefensins, particularly as sulfonate-containing metabolites are not common in animals. We conservatively estimate that compound **1** represents a pool of 1 million ton in European soils alone (assuming 500 kg ha^−1^ earthworm biomass for forest and farmland^26^). Ultimately, given the global abundance of earthworms, these metabolites may play a key ecological role in contributing to the turnover of >10^10^ ton of plant carbon per annum. Furthermore, this pool is undoubtedly rapidly turned over: drilodefensins reduce in concentration along the earthworm gut, and are not detectable in earthworm castings (Fig. 2), indicating the presence of a recycling system. Hence, an important question that is yet to be resolved is to determine exactly what happens to the surfactant complexes—are the undigested polyphenols simply released from the complex in the posterior part of the gut for excretion? We also do not know if drilodefensin molecules are recycled by transport back to the foregut, or if they are enzymatically degraded and the nutrients recovered.

Ultimately, these metabolites provide a mechanism for earthworms to cope with high levels of polyphenols in their diet—essential for detritivores that contribute so largely to turning over plant-derived material in soils. This gives earthworms the ability to populate many habitats, including those with polyphenol-rich plant litter, such as woodland soils.

## Methods

### Feeding experiment

Earthworms used for the feeding experiment were taken from a culture originally established from a field-collected population. The culture was kept outdoors in large boxes under a cover in a 1:1:1 soil:bark:compost medium for 6 months before the test. The feeding test itself was conducted in a clay loam soil with a pH of 7.1 and a 5% organic matter content that was further amended with 3% organic matter. Each of the 10 replicate containers per treatment contained 250 g dry weight of this soil wetted to 50% water holding capacity. A single adult *Lumbricus rubellus* was added to each replicate. The food sources were comprised of one control diet (oats only), two with increased lipid content (amended with either olive oil or animal fat) and two with high polyphenol levels (amended with either a pure standard of tannic acid, or oak leaves), and were made up as follows: oats only (60 g dry weight oats+100 ml H_2_O); oats+vegetable oil (60 g dry weight oats+12 g olive oil+90 ml H_2_0); oats+animal fat (60 g dry weight oats+12 g lard+90 ml H_2_O); oats+tannic acid (60 g dry weight oats+6 g tannic acid+100 ml H_2_O to give a final concentration of 100 mg g^−1^ tannic acid in oats); oak leaves (50 g chopped oak leaves (freshly fallen)+200 ml H_2_O). At the start of the experiments individual earthworms were fed either 8.7 g of wetted oats (giving 3 g dry weight per replicate for all oats treatments), or 7.5 g of oak leaves (giving 1.5 g dry weight per replicate). After 2 weeks of exposure, the earthworms were retrieved from the containers, weighed and immediately frozen in liquid nitrogen. All worms used in the experiment survived the exposure resulting in a full set of 10 earthworms per treatment for analysis.

Whole earthworms were extracted in 7 ml tubes containing 2.8 mm zirconium oxide beads (Precellys Ceramic Kit, Peqlab, Germany) and a solvent mixture of acetonitrile, methanol, water (2:2:1) (ref. 19), with the volume adjusted to the whole worm weight. Extraction and tissue disruption was done in a Precellys Dual bead beater (Bertin Technologies) at 5,000 r.p.m. for 40 s. The samples were subsequently centrifuged (3,000*g*, 2 min, 4 °C) and supernatants were transferred into new tubes. All steps were performed on ice to prevent enzymatic degradation. For final analysis, 50 μl of supernatant were diluted to 200 μl with the extraction solvent and analysed by UPLC with ultraviolet detection.

### Earthworm field populations for drilodefensin quantification

Adult *L. rubellus* were collected and analysed for drilodefensin (compound **1**) quantification from 13 grassland sites, 3 mine sites and 1 woodland site in the United Kingdom. The details of the sampling procedures and a list of the site characteristics (set 1) have been previously reported^27^.

### UPLC-ultraviolet detection of alkylfuransulfonic acids

A Waters Acquity UPLC system with a ultraviolet/visible absorbance photodiode array detector (monitoring *A*_230_) using an Acquity HSS T3 1.8 μm column (2.1 × 30 mm) was used for the separation and detection of alkylfuransulfonic acids. Elution was performed at 0.4 ml min^−1^ in a gradient of solvents A (0.1% formic acid in water) and B (0.1% formic acid in isopropanol:acetonitrile 70:30 vol/vol): 20% B increasing to 60% B over 1 min, further increase to 99% B over 0.5 min, isocratic for 0.5 min, B was decreased to 80% over 1 min and re-equilibrating over 1 min. For analysis MarkerLynx Version 4.1 (Waters, Milford, MA, USA) was used.

### Analysis of excreted fluids and gut content

Samples for the analysis of excreted fluids and gut content were prepared as followed. Single adult *Lumbricus terrestris* earthworms were placed in a 9 cm Petri dish containing a Whatman No.1 filter paper disc with 3 ml water to keep them moist. These were kept in the dark at 12 °C for 3 days, afterwards the filter with fluids and gut content was added to 10 ml water and mixed thoroughly and subsequently centrifuged in 15 ml tubes for 5 min at 3,000 r.p.m. Supernatants from 10 worm samples were combined and dried in a vacuum concentrator (Eppendorf, Cambridge, UK). Dried samples were resuspended in NMR buffer and analysed by ^1^H NMR.

### Determination of elemental composition

Adult *L. terrestris* earthworms were frozen in liquid nitrogen and the tissue was powdered under liquid nitrogen conditions using a cryogenic impact mill (freezer mill 6870, SPEX, Stanmore, UK). One aliquot of the frozen powder was directly prepared for the analysis of elemental composition, another aliquot was extracted with a solvent mixture of acetonitrile, methanol, water (2:2:1). The extract was centrifuged (3,000*g*, 2 min, 4 °C), afterwards the whole pellet containing cell debris and precipitated proteins was prepared for elemental analysis (‘non-soluble' sulfur). The supernatant (‘soluble sulfur') was further separated via solid-phase extraction to obtain a fraction solely consisting of drilodefensins and a fraction of other soluble compounds. Each fraction was freeze-dried and the powder sent for elemental analysis (C, N, S) by a commercial service (Elemental Microanalysis, Okehampton, UK). The solid-phase extraction used a mixed-mode reversed-phase/weak anion exchange column (Strata-X-AW, Phenomenex, UK) using acidic methanol (0.05% trifluoroacetic acid vol/vol) for eluting other soluble compounds, followed by basic methanol (2.5% ammonium hydroxide vol/vol) for eluting the drilodefensins.

### Tissue material for mass spectrometry imaging

Adult *L. rubellus* were collected from laboratory cultures maintained at the Centre for Ecology and Hydrology, Wallingford, UK, washed with distilled water and depurated at 12 °C in the dark for at least 3 days. Worms were snap-frozen in liquid-nitrogen-cooled isopentane, and stored at −80 °C until further processing.

### Cryosectioning

Frozen earthworms were cut into ∼0.5 cm parts with a precooled scalpel, transferred to a cryostat cooled to −23 °C and fixed to a sample plate with a droplet of optimal cutting temperature medium; for details see Wroblewski *et al*.^28^. The tissue part for mass spectrometric analysis was not covered with optimal cutting temperature. Cross-sections with 15 μm thickness were cut from parts of the head region, the clitellum and the tail region. These were immediately transferred onto glass slides with an artist brush and thaw mounted. Polylysine-coated glass slides (Glass Slides for MALDI imaging, Bruker Daltonics) were used for MALDI-MS experiments and plain glass slides for TOF-SIMS experiments, respectively. The tissue sections were dried in a desiccator connected to a membrane pump for several hours until completely dry. Optical images were taken with a standard light microscope connected to a digital camera or with the MIRAX desk digital slide scanner (Zeiss, Germany). An Olympus VS120-S virtual slide scanning system was used to scan slides containing samples for DESI-MS and TOF-SIMS. During all transfers precautions were taken against tissue rehydration from room atmosphere.

### MALDI-MS imaging

For homogeneous matrix deposition, the ImagePrep instrument (Bruker Daltonics, Germany) was used. This sensor-controlled vibrational vaporization instrument was set according to manufacturer's instructions for the application of alpha-cyano-4-hydroxycinnamic acid (HCCA; 7 g l^−1^ HCCA matrix in water/acetonitrile/trifluoroacetic acid=49.9/49.9/0.2). All chemicals used were obtained in high purity from Sigma, except HCCA (Bruker Daltonics) and solutions were prepared after established protocols^29^.

For MALDI-MS measurements, the prepared slides were mounted into a Slide Adapter (Bruker Daltonics) and loaded into the dual source of a 12 T Fourier transform ion cyclotron resonance, (FTICR)-MS (solariX, Bruker Daltonics). MALDI images were acquired with a *x*–*y*-raster width of 50 μm using smartbeam II laser optics with a 30 μm laser focus. For each pixel a single scan was recorded using the ions generated by 300 laser shots. The laser was pulsed at 1 kHz and the ions were accumulated externally (hexapole) before being transferred into the ICR cell for a single scan. For each scan 1 M data were acquired for the mass range 130–1,500 followed by a single zero filling and a sine apodization function. For a given imaging experiment up to 5,854 pixels were acquired. External calibration was carried out using arginine clusters in electrospray mode. This provided a mass measurement accuracy of <1 p.p.m. over the *m*/*z* range of interest.

After MS acquisition removal of the matrix and haematoxylin and eosin (H&E) staining was performed according to standard protocols and optical images were recorded using a MIRAX desk digital slide scanner (Zeiss, Germany). All images, MALDI-MS image, unstained tissue scan and H&E stained tissue scan were combined and co-registered using the flexImaging software Version 3.0 (Bruker Daltonics). Mass spectral information was extracted using a width of 2 mDa.

### TOF-SIMS imaging

TOF-SIMS was carried out using an IONTOF ToF-SIMS^5^ instrument. The instrument is a dual beam system and can be operated in a non-destructive static mode for ion mapping and mass spectrometry or in a destructive ‘dynamic' mode for depth profiling.

Analyses were carried out using the Bi_3_^+^ ion beam in the burst alignment mode to give optimal lateral resolution for ion mapping. In all cases, negative secondary ions were collected and charge compensation of the sample surface was supplied by an electron flood gun (20 eV). Ion beam doses during the ion mapping were maintained below the static limit to ensure the sample surface was not sputtered.

Two different modes of ion mapping were carried out: large ion maps were collected by continuously moving the sample stage over a selected region of interest and stitching together individual analysis areas of 0.5 mm^2^. This is known as a ‘stitched' image. To map the whole sample an area of 5 × 5 mm^2^ was selected (containing 3,500 × 3,500 pixels). Smaller ion maps were collected by rastering the ion beam over a chosen field of view and selected number of pixels, in this case areas of 500 × 500 μm^2^ with 1,024 × 1,024 pixels were used for more specific regions of interest on the sample.

Following MS acquisition, tissues were stained with the H&E staining protocol and optical images were taken with above mentioned microscopes and cameras.

Identification of selected metabolites, for example, compound **1** was done by spotting pure compound solutions onto glass slides and analysing the respective mass spectra under given tissue imaging conditions.

### DESI-MS imaging

DESI experiments were carried out using a home-built motorized DESI ion source as described elsewhere^30^ mounted on a Thermo Orbitrap Discovery XL instrument Thermo Scientific, Bremen, Germany) operated in negative ion mode. The distance between DESI sprayer and sample surface was set to 2 mm; the distance between the sprayer and the inlet capillary was 14 mm and the distance between inlet capillary and sample was set to <<1 mm. Nitrogen (BOC Group, Guildford, UK) was used as nebulizing gas at a pressure of 7 bar. Methanol/water (95:5 vol/vol) was used as spray solvent at −4.5 kV and a flow rate of 1.5 μl min^−1^. Ultra gradient solvents were purchased from Romil Ltd., Cambridge, UK. Spray angle and collection angle were set to 80° and 10°, respectively. Spatial resolution for the imaging experiment was set to 40 μm.

Individual line scans were recorded bin the mass range *m*/*z* 150–1,000 and raw files were subsequently converted into.imzML files using the imzML Converter Version 1.1.4.5. Single ion images and RGB images were generated using MSiReader Version 0.05 (ref. 31) with linear interpolation (order 1) and 0.005 Da bin size.

### Direct infusion nanospray ionisation (NSI)-MS of earthworm extracts

Direct infusion nanospray ionization experiments were carried out using a home-built nanospray ion source mounted on a Thermo Orbitrap Discovery instrument. The instrument was operated in negative ion mode and covering a mass range of *m*/*z* 70–300. Nanospray experiments were performed to obtain tandem mass spectra of high mass resolution (*R*=30,000 at *m*/*z* 400) and accuracy (<3 p.p.m.) to determine structural formulas of the generated fragments. −1 kV high voltage were applied to gold-coated 5 μm ID nanospray needles (obtained from DNU-MS GbR, Berlin, Germany) to nebulize the extracts for mass spectrometric analysis.

### Mass spectrometer parameters for DESI-MS and NSI-MS

DESI imaging experiments were performed using the Fourier transform-MS analyzer of the Orbitrap Discovery XL hybrid instrument. High-resolution full scan mass spectra were acquired over the mass range *m*/*z* 150–2000 using a fixed injection time of 1,000 ms and 1 microscan. Tube lens voltage and capillary voltage were set to −100 and −20 V, respectively. Inlet capillary temperature was set to 250 °C.

For nanospray ionization experiments, mass spectra were acquired over the mass range *m*/*z* 70–300 using a home-built nanospray ionization source operated at 1 kV spray voltage. Maximum injection was set to 1,000 ms and one microscan was acquired per mass spectrum (automatic gain control active). Capillary temperature and capillary voltage were kept constant at 250 °C and −20 V, respectively, while the tube lens voltage was set to −80 V. All analyses were performed in negative ion mode.

### Three-dimensional model of IMS data

A three-dimensional (3D) model of earthworm anatomy based on open-access μCT data (specimen #MCZ_24805, http://dx.doi.org/10.5524/100092)^32^,^33^, including volume rendering, clipping and cropping was created using version 2.4 of the free 3D imaging software Drishti^34^ (http://sf.anu.edu.au/Vizlab/drishti/index.shtml). Mass spectrometry imaging data was registered into this model by locating the planes of sectioning and correlating histological features.

### Synthesis of compound **1** and **2**

Compounds **1** and **2** were synthetized after the scheme shown in Supplementary Fig. 3a. Masked 4-hydroxy-ynone **8**, obtained in three standard transformations from commercially available heptanal **7**, proved to be a viable substrate for hydrobromination and aromatization *in situ*, in a modification of Obrecht's general methodology^35^. Metal–halogen exchange and sulfonylation^36^ gave synthetic **1** as a caramel-coloured solid following column chromatography and treatment with activated charcoal to remove coloured impurities. 2-ethyl-5-hexylfuran **10**, resulting from protonation of the intermediate organolithium compound (X=Li), was always formed as a significant side product but was separated by extraction during the work-up. Compound **2** was made analogously from commercially available oct-1-yn-3-ol. Reagents and conditions: (i) HC≡CMgBr, THF, 0 °C to room temperature; (ii) DHP, PPTS, CH2Cl2; (iii) EtMgBr, THF then *N*-methoxy-*N*-methylpropionamide, 0 °C to room temperature (84% from **7**); (iv) HBr (4.0 M, aqueous), toluene, 65 °C (75%); (v) BuLi, hexanes/THF, –78 °C then Me3N·SO3, –78 °C to room temperature (31%) (THF, tetrahydrofuran; DHP, dihydropyran; PPTS, pyridinium *para*toluenesulfonate; THP,tetrahydropyranyl).

### Chemical characterization of compound **1** and **2**

Synthetic products of compound **1** and **2** were characterized analytically: Obtained values for compound **1**:^1^H NMR (400 MHz, CD_3_OD): *δ* 6.14 (t, *J*=1.0 Hz, 1H), 2.87 (t, *J*=7.5 Hz, 2H), 2.57 (qd, *J*=7.5. 1.0 Hz, 2H), 1.65 (quin, *J*=7.5 Hz, 2H), 1.26–1.40 (m, 6H), 1.20 (t, *J*=7.5 Hz, 3H), 0.90 (t, *J*=7.5 Hz, 3H); ^13^C NMR (101 MHz, CD_3_OD): *δ* 156.0, 154.8, 126.3, 105.3, 32.8, 30.1, 29.4, 27.6, 23.7, 22.0, 14.4, 12.5; IR (thin film): 3,425 (m), 1,369 (w); HRMS (ESI^–^) *m/z*: found 259.1009; calcd. for C_12_H_19_O_4_S^–^ [(M–H^+^)^–^] requires 259.1010. Obtained values for compound **2**:^1^H NMR (400 MHz, CD_3_OD): *δ* 6.11 (t, *J*=1.0 Hz, 1H), 2.83 (t, *J*=7.5 Hz, 2H), 2.53 (qd, *J*=7.5. 1.0 Hz, 2H), 1.62 (quin, *J*=7.5 Hz, 2H), 1.26–1.35 (m, 4H), 1.16 (t, *J*=7.5 Hz, 3H), 0.87 (t, *J*=7.5 Hz); ^13^C NMR (101 MHz, CD_3_OD): *δ* 156.0, 154.8, 126.4, 105.3, 32.6, 29.1, 27.6, 23.5, 22.0, 14.4, 12.5; IR (thin film): 2,970 (w), 1,739 (s), 1,366 (m), 1,217 (s) cm^–1^; HRMS (ESI^–^) *m/z*: found 245.0851; calcd. for C_11_H_17_O_4_S^–^ [(M–H^+^)^–^] 245.0853. ^1^H NMR and ^13^C NMR spectra for compound **1** and **2** can be found in Supplementary Figs 9 and 10.

### Lumbrokinase activity assay

Earthworm gut protease activity can be monitored using chromogenic substrates^37^. The fibrinolytic active enzyme protease EfP-III, also known as lumbrokinase, was purchased from Nutricology (USA) and its activity was assayed using *N*-benzoyl-L-arginine ethyl ester (BAEE) as substrate. Mixtures of lumbrokinase in buffer with the addition of different amounts of SDS or compound **1** or **2** were incubated for 10 min at room temperature before adding to the BAEE solution. For hydrolysis of a 0.25 mM BAEE solution (67 mM Na_2_HPO_4_ buffer, pH 7.0), 250 μg lumbrokinase were added and the absorbance was measured at 253 nm over 5 min. Activities were compared using the slope of the enzymatic reaction within the first 150 s.

### Protein precipitation assay

Tannic acid precipitates bovine serum albumin (BSA) from a buffer solution in a concentration-dependent fashion. The ability of different concentrations of SDS or compound **1** or **2** to reduce BSA precipitation were monitored. A BSA solution (16 μg ml^−1^ in buffer containing 0.2 M acetate, 0.17 M NaCl, pH4.9) was mixed with defined amounts of surfactant or plain buffer, and incubated for 10 min at room temperature. A 0.1 mM tannic acid solution was added to the incubations to a final concentration of 0.018 mM, vortexed vigorously and incubated for a further 30 min. The mixture was centrifuged at 4 °C for 20 min at 25,000*g*. The supernatants were removed by aspiration, and the precipitates were gently washed with 200 μl of buffer and were centrifuged again for 1 min. The samples were again aspirated and the precipitates analysed. Precipitated protein was determined using the Amido Black assay, as this is less sensitive to interferences from polyphenols than other common protein assays^8^,^38^. Precipitates were redissolved in 1% SDS solution (0.05 M Tris, pH 7.5) and a trifluoracetic acid solution was added, incubated for 5 min at room temperature. Solutions were vacuum filtered onto a 0.1 μm nitrocellulose membrane; microtubes were rinsed with diluted TFA solution. The filter membranes were dried and afterwards stained for 10 min with Amido Black solution (1 g l^−1^ in water). After destaining and dissolution of the dye following the method of Weiss and Bisson^38^, *A*_630_ values were compared to a BSA standard curve prepared with the same procedure.

### Cholesterol solubilization assay

To measure the sterol solubilizing power of a given compound, dried mixtures with cholesterol (25 mM) and either cholic acid, SDS or compound **1** or **2** in different amounts were hydrated in 1 ml of 0.10 M phosphate buffer, pH 7.0, mixed, shaken for 2 h at room temperature and centrifuged at 16,000*g*; the cholesterol concentration in the supernatant was determined by gas chromatography–mass spectrometry.

## Additional information

**How to cite this article:** Liebeke, M. *et al*. Unique metabolites protect earthworms against plant polyphenols. *Nat. Commun.* 6:7869 doi: 10.1038/ncomms8869 (2015).

## Supplementary Material

Supplementary Information Supplementary Figures 1-10, Supplementary Tables 1-2 and Supplementary References
