# Supplementary material for: Unique metabolites protect earthworms against plant polyphenols
Source: Nat Commun. 2015 Aug 4;6:7869. doi: 10.1038/ncomms8869 (PMC4532835; doi:10.1038/ncomms8869)
Supplement: Supplementary Information — Supplementary Figures 1-10, Supplementary Tables 1-2 and Supplementary References [file ncomms8869-s1.pdf]

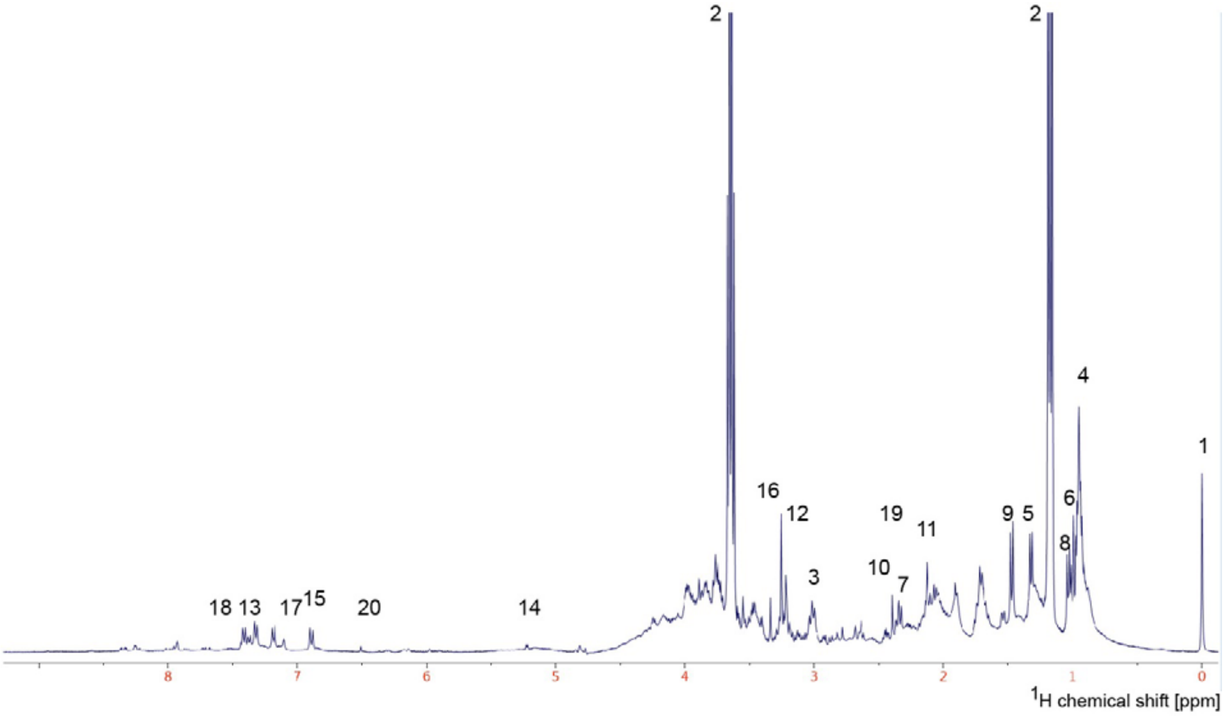

Suppl. Figure 1. Metabolite profile of gut fluid from earthworm *Lumbricus rubellus*.

Adult worms were anesthetized with ethanol solution and dissected to sample gut fluid with a syringe. Obtained gut fluid from fore- and midgut was centrifuged and the supernatant was mixed with H_2O/D_2O based NMR buffer and ^1H-NMR spectra were recorded. Major peaks identified by matching spectra from pure compounds: 1-DSS (4,4-dimethyl-4-silapentane-1-sulfonic acid as internal standard), 2-ethanol, 3-lysine, 4-leucine, 5-lactate, 6-isoleucine, 7-glutamine, 8-valine, 9-alanine, 10-glutamate, 11-methionine, 12-phosphoethanolamine, 13-phenylalanine, 14-glucose, 15-tyrosine, 16-betaine, 17-histidine, 18-tryptophan, 19-succinate, 20-fumarate

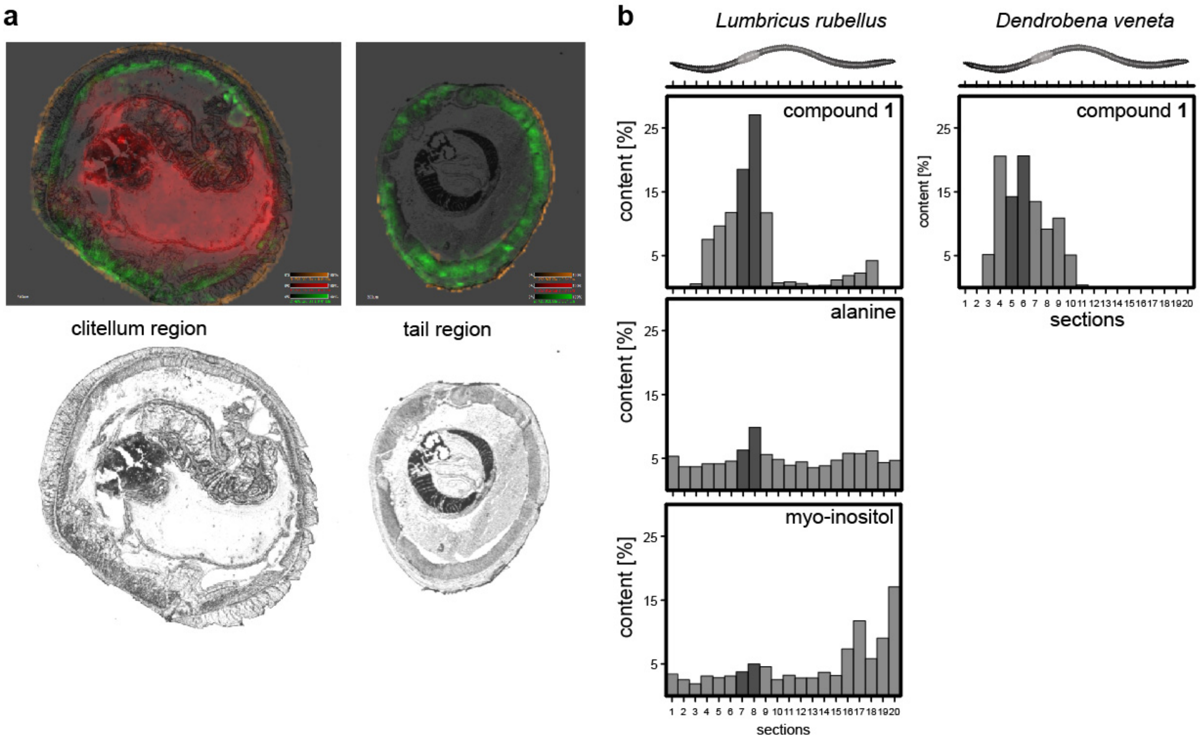

Suppl. Figure 2. Longitudinal whole-organism metabolite distribution.
a, MALDI-MS images for earthworm cross-sections.
MALDI-MS image and optical image of a section through the region of the clitellum and the tail region. Tissue sections analyzed by MALDI-MS (neg. MS mode) through the tail region of an adult *L. rubellus* show no signals of compound **1** [259.01 Da], whereas **1** is high abundant in the clitellum cross section. Signals for [426.02] (ADP) and [259.90] (unknown compound) show in both sections a similar distribution in the outer tissue rings. Mass traces are displayed as different colors.
b, Quantitative metabolite distribution in earthworms was determined by ^{1}H NMR, adult worms were sectioned into equal parts (20 parts, each ~0.3cm) from head to tail, the frozen tissue was cut with a cooled scalpel on a liquid N_2 cooled block of metal. Tissue extracts were prepared and analyzed by ^{1}H NMR. Selected metabolite distributions are shown for *Lumbricus rubellus* and *Dendrobena veneta*. Dark bars represent data from sections in the region of the earthworm clitellum.

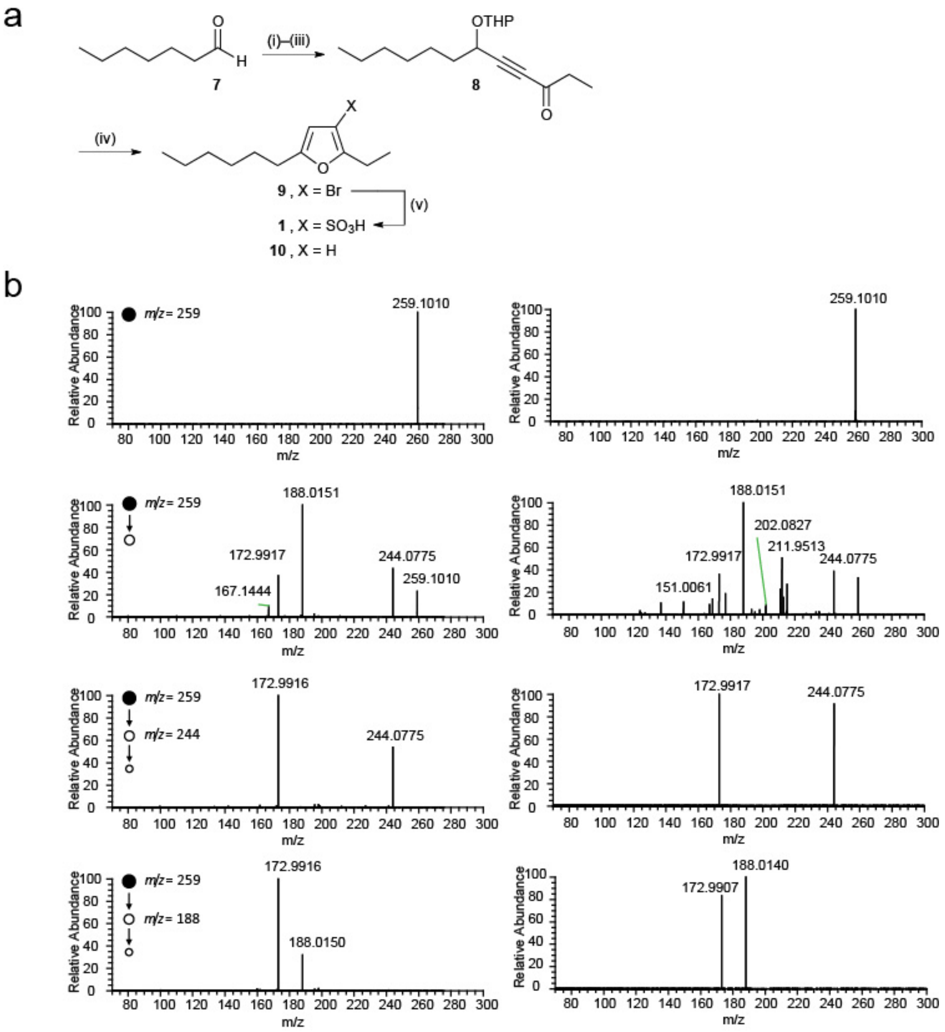

Suppl. Figure 3. Synthesis scheme for compound 1 and mass spectral characterization
a, A synthetic sample of compound **1** and **2** was obtained using the route summarized here.
b, MS_n characterization of compound **1** from biological purification (left panel) and synthetic origin (right panel). Compound **1** was purified from earthworms. Worm tissue was mechanically disrupted and extracted with a mixture of acetonitrile, methanol and water (2:2:1, vol./vol.). The obtained extract was acidified with trifluoroacetic acid and further purified with a weak anion exchange solid-phase extraction column, followed by separation on a C_{18} SPE column.

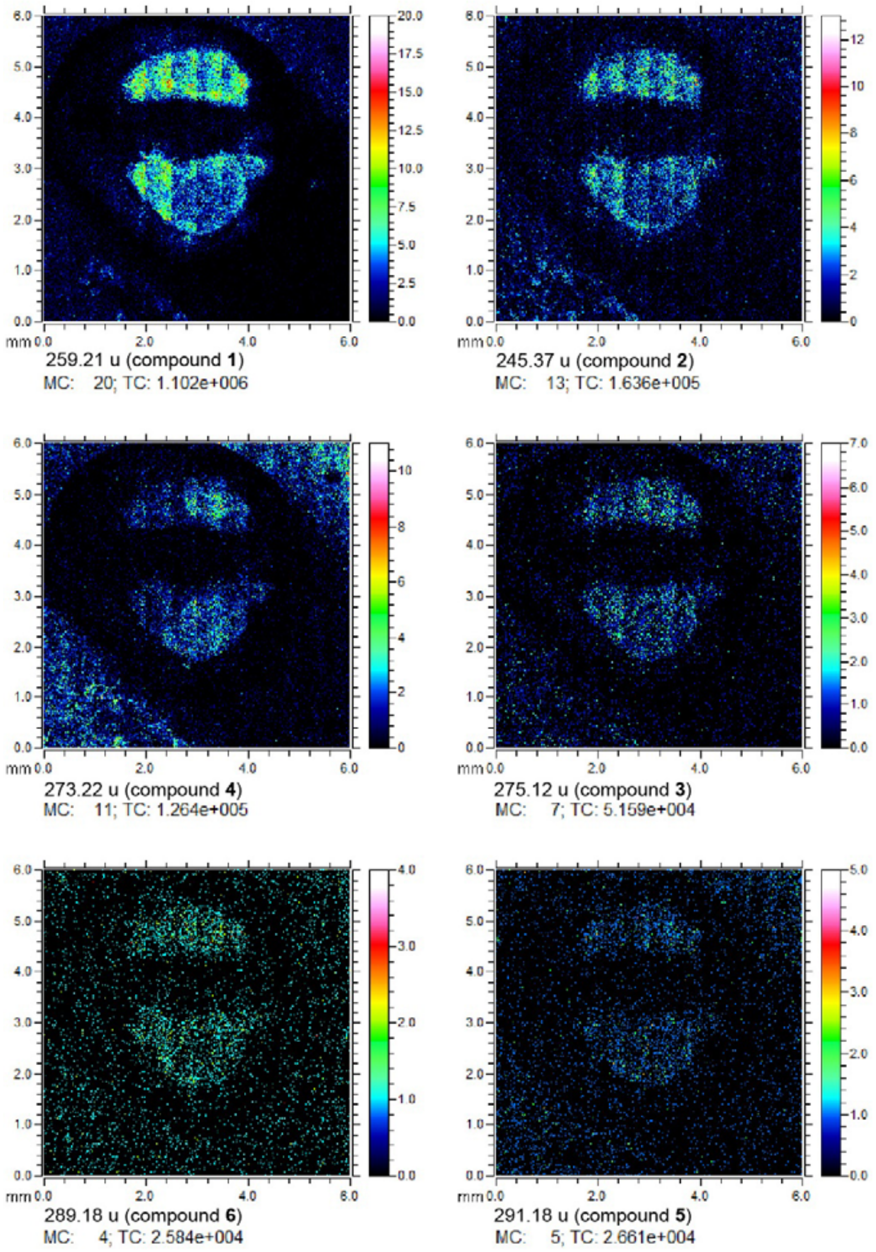

Suppl. Figure 4. Co-localized ions in the earthworm gut lumen
TOF-SIMS mass spectral data on a cross-section of earthworm tissue showed abundant co-localized ions with the major compound of interest (compound **1**). Color-bar indicates high and low ion abundances on the section, TC (total ion count), MC (maximum ion count).

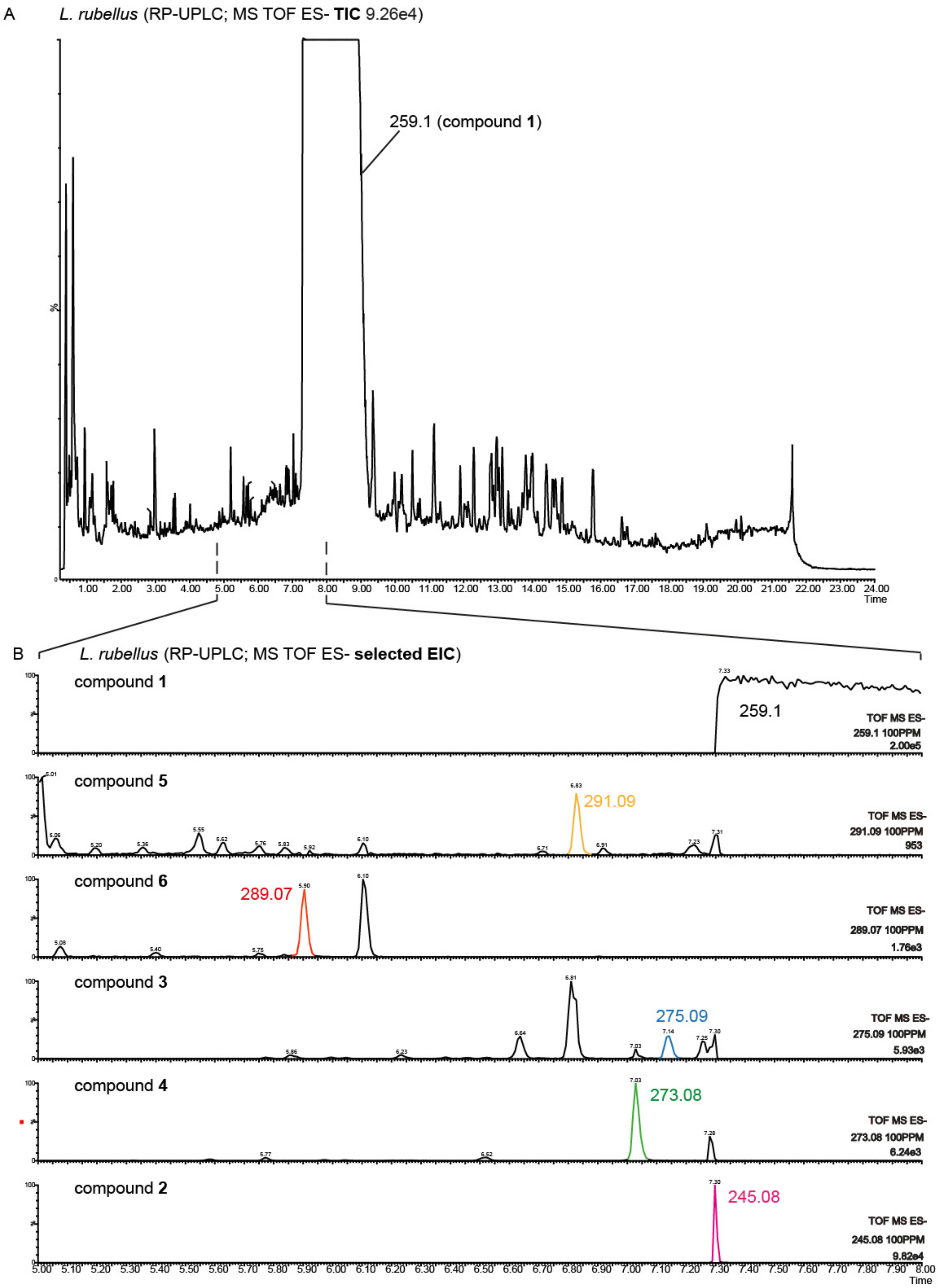

Suppl. Figure 5. Detection of compounds 1-6 in whole tissue extracts by UPLCMS
Whole worm extract (*L. rubellus*) was analyzed by UPLC-MS for the presence of compounds **1-6**. Extracted ion chromatograms are shown for each compound.

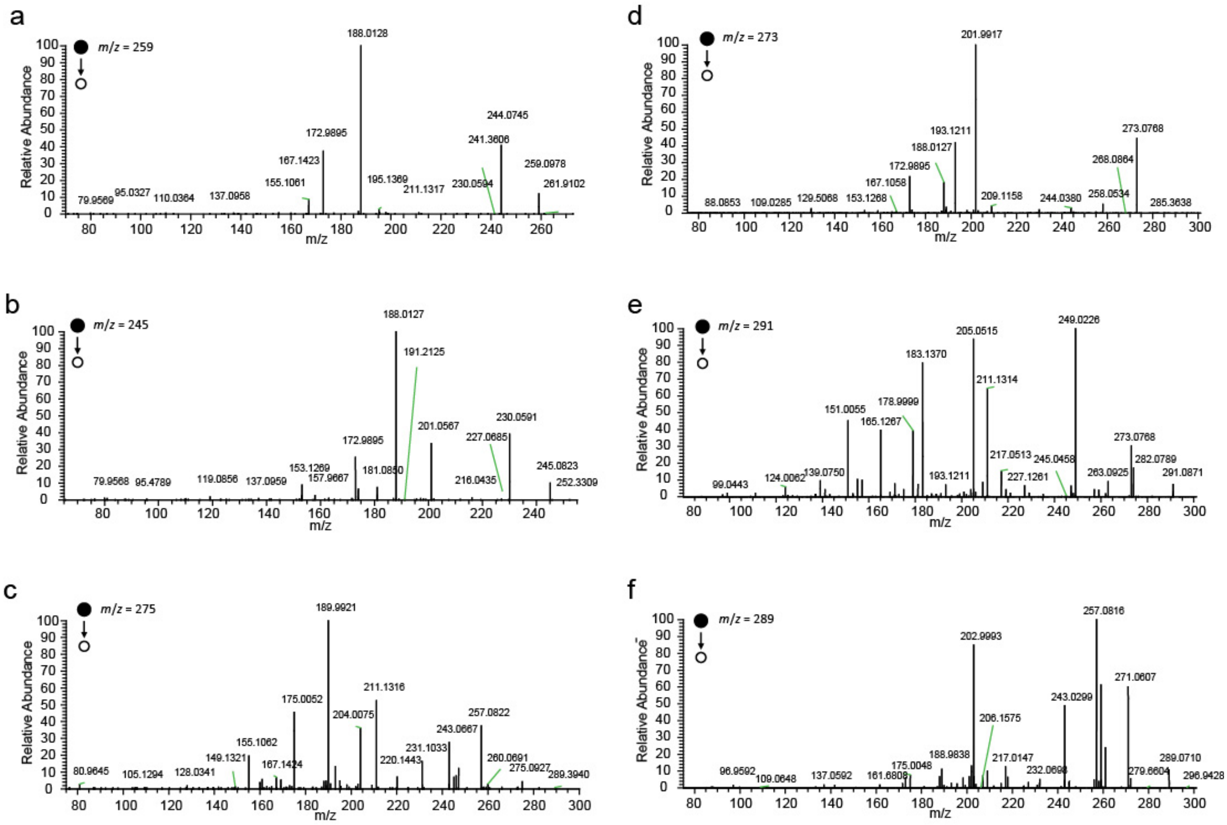

Suppl. Figure 6. HR MS and MS_n spectra of compounds (1-6)

Mass spectra and calculated sum formula are displayed from **a**) to **f**) for compounds **1** to **6**, respectively. Tandem mass spectrometry experiments were performed using two methods, by DESI MS/MS directly on the section using parameters described in material and methods and by nanospray ionization (NSI) of earthworm extracts (prepared as described above, including an additional filtration through a 3kDa filter membrane). For the NSI experiments, 10 µL extract were inserted into nanospray needles (5 µm ID, 55 mm length, obtained from DNU-MS GbR, Berlin, Germany) and analyzed using a home-built NSI source. Both DESI and NSI experiments were carried out using an Orbitrap Discovery Hybrid instrument (Thermo Scientific GmbH, Bremen, Germany). All spectra were recorded in negative ion mode using the Orbitrap mass analyzer. Additional instrumental and source parameters are summarized in material and methods.

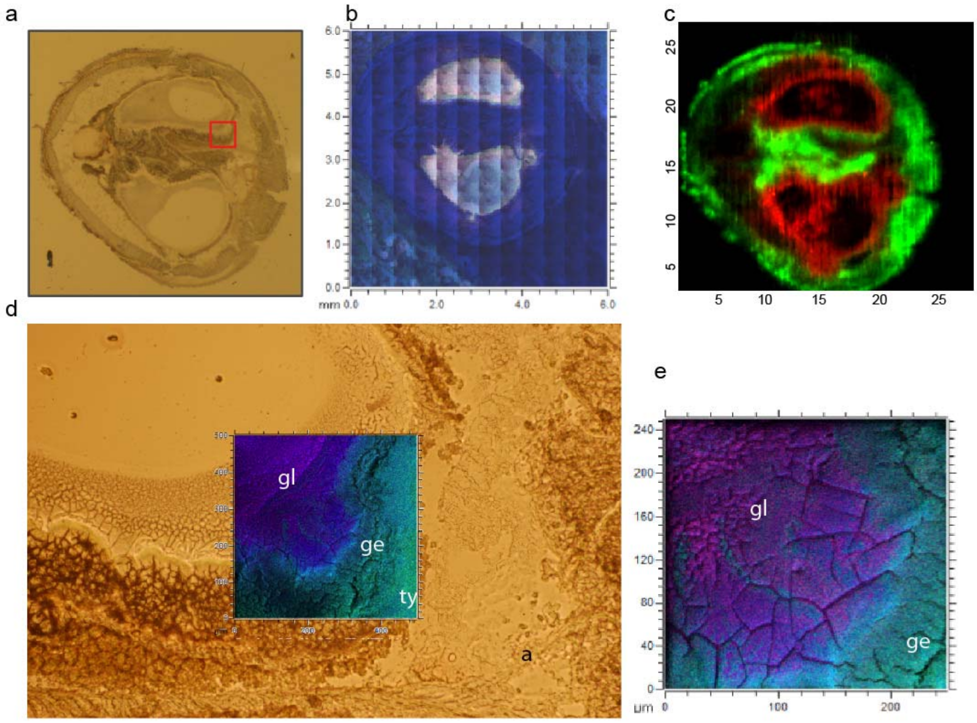

Suppl. Figure 7. TOF-SIMS and DESI-MS analysis of earthworm cross-section
Tissue section through the clitellum region of an adult *L. rubellus* analyzed by MSI. **a**, optical image for comparison and signals of compound **1** observable by **b,** TOF-SIMS analysis and **c,** DESI-MS (in both cases prominent [M-H+] 259.1 Da ion highlighted with red color). DESIMS analysis was performed directly after TOF-SIMS analysis on the same section. Both MS imaging techniques show similar distributions of compound **1**, slight differences in intensity of ion counts may appear based on different ionization and pressure conditions. Overlay of TOF-SIMS image (500x500µm) and optical image is shown in **d)** to verify the distribution of **1** in the gut lumen and not the adjacent gut epithelium cells. A further TOF-SIMS image with higher spatial resolution (225x250µm) from the region of gut lumen and gut epithelium is displayed in panel **e)**.

class	family	species	habitat	
Clitellata	Lumbricidae	*Allolobophora chlorotica*	soil	●
		Aporrectodea tuberculata	soil	●
		Aporrectodea caligonosa	soil	●
		Eisenia fetida	soil	●
		Eisenia veneta	soil	●
		Lumbricus rubellus	soil	●
		Lumbricus terrestris	soil	●
	Megascolecidae	*Amynthas rodericensis*	soil	●
		Amynthas corticis	soil	●
		Amynthas gracilis	soil	●
		Pithemera bicincta	soil	●
	Glossoscolecidae	*Pontoscolex corethrurus*	soil	●
	Enchytraeidae	*Enchytraeus buchholzi*	soil	●
	Hirudinidae	*Hirudo medicinalis*	limnic	●
	Tubificidae	*Tubifex Tubifex*	limnic	●
		Olavius algarvensis	marine sediment	●
	Lumbriculidae	*Lumbriculus variegatus*	limnic	●

Suppl. Figure 8. Invertebrate species screened for drilodefensins.
Diverse invertebrates covering 7 families were screened for dialkylfuransulfonic acids. Only earthworms contained detectable amounts of compound **1** (green circle), whereas the other species showed no trace of **1** (red circles). *Lumbriculus variegatus*, *Dendrobena veneta*, and *Hirudo medicinalis* were purchased from Blades Biological Ltd. (Edenbridge, UK). *Lumbricus rubellus*, *Lumbricus terrestris, Aporrectodea tuberculata, Allolobophora chlorotica*, and *Enchytraeus buchholzi* were sourced from existing cultures at CEH Wallingford, UK (or else collected on site there). *Olavius algarvensis* (Tubificidae) was provided by M. Kleiner (MPI, Bremen, Germany). *Tubifex tubifex* (Tubificidae) was purchased from an aquarium supplier. *Amynthas rodericensis, Amynthas corticis*, *Pithemera bicincta* (all Megascolecidae), and *Pontoscolex corethrurus* (Glossoscolecidae) as well as *Amynthas gracilis* (Megascolecidae) worms were collected in the Royal Botanic Gardens, Kew, London (UK) and from the island of Furnas (Portugal) in the case of *A. gracilis* samples. After collection all organisms were snap-frozen, manually ground under liquid N_2 using a mortar and pestle, and the ground tissue powder extracted with a mixture of acetonitrile, methanol, water (2:2:1) and analyzed by ^{1}H NMR using previously reported methods.[1]

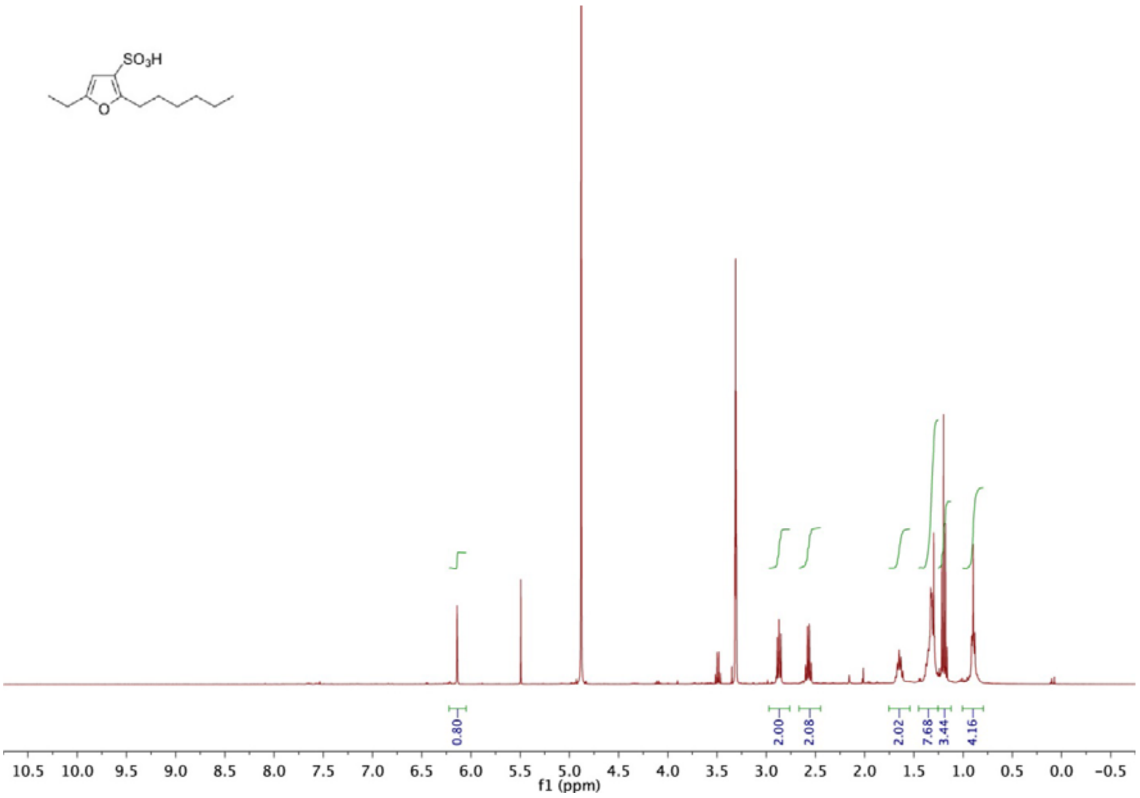

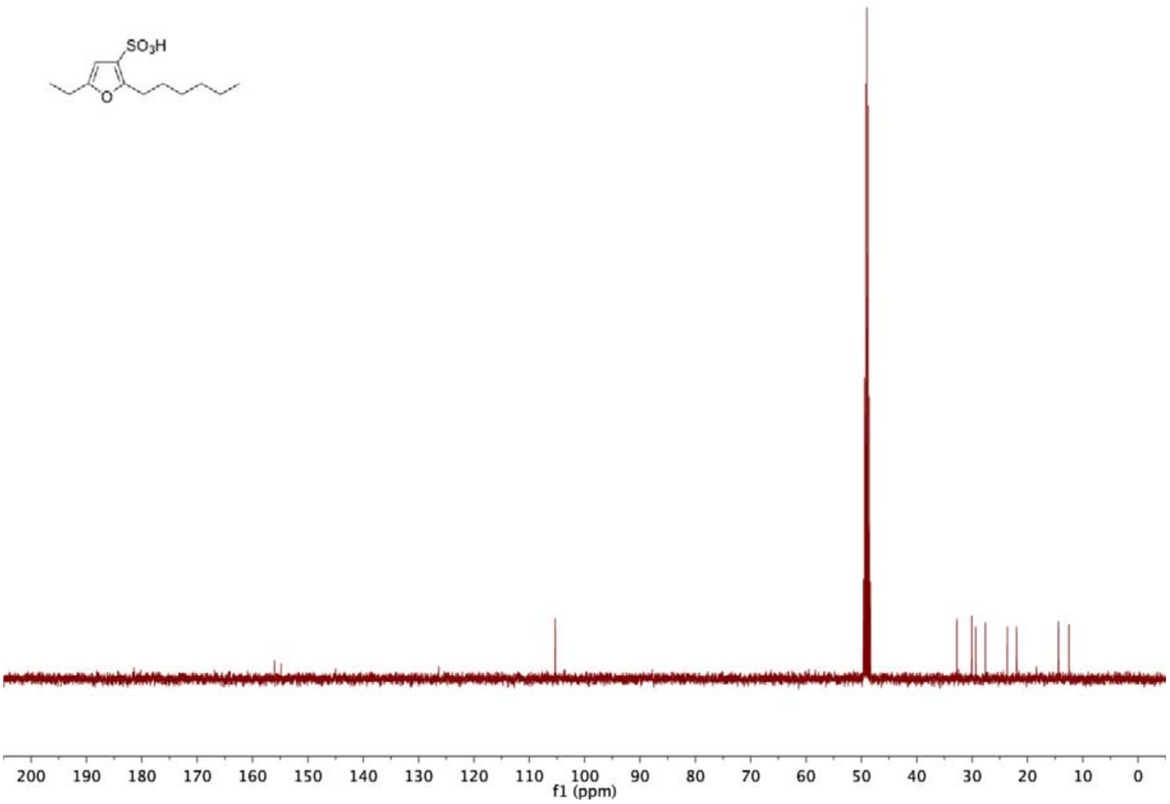

Suppl. Figure 9. ^{1}H NMR (400 MHz, CD_3OD) and ^{13}C NMR (101 MHz, CD_3OD) spectra for compound **1.**

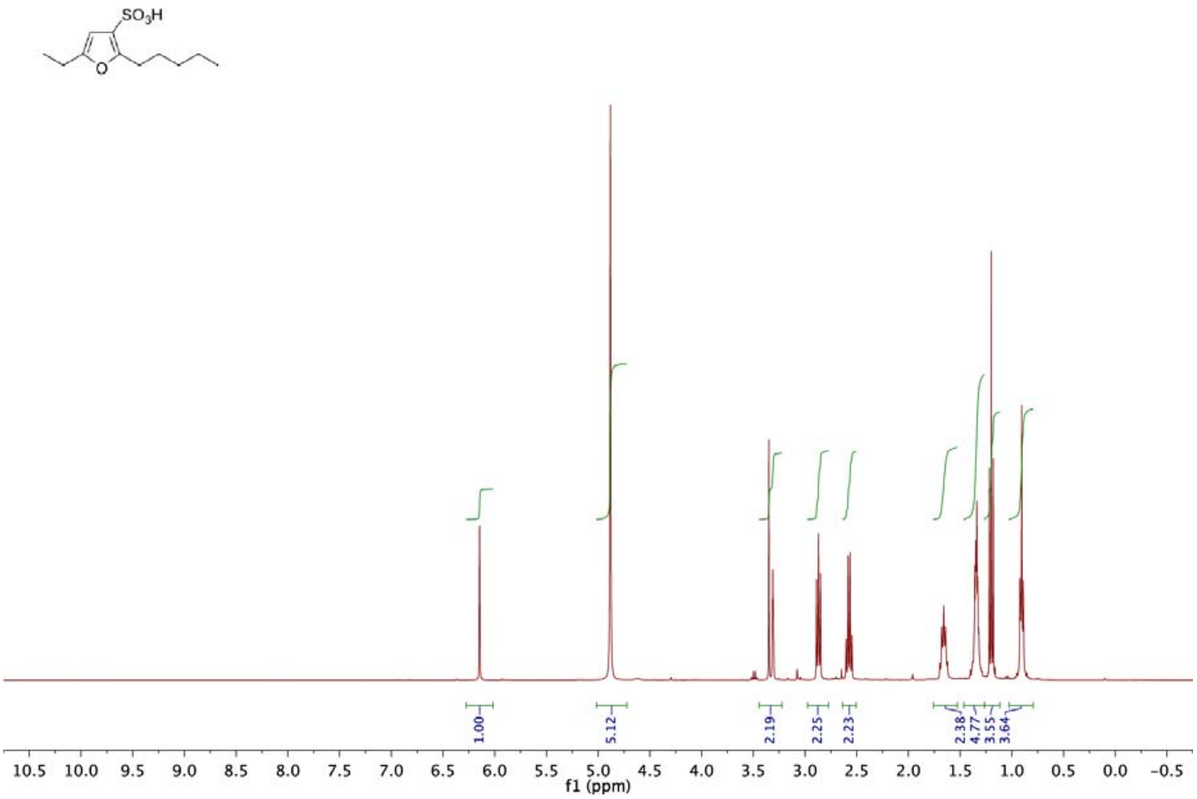

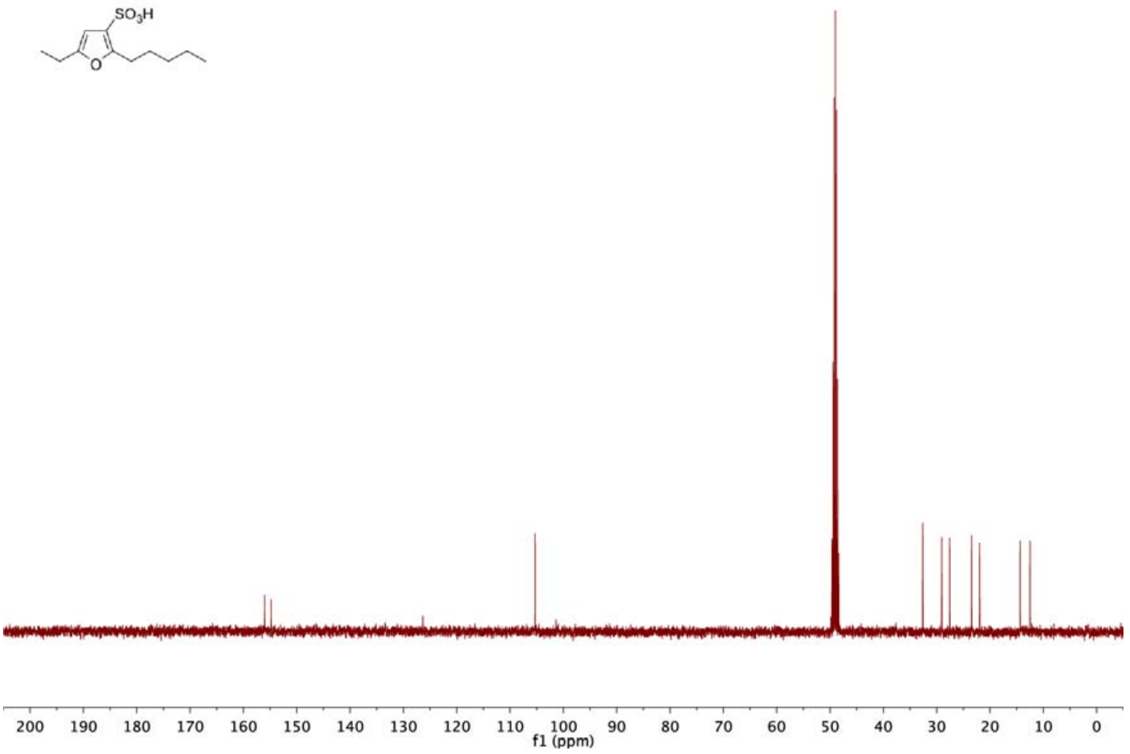

Suppl. Figure 10. ^{1}H NMR (400 MHz, CD_3OD) and ^{13}C NMR (101 MHz, CD_3OD) spectra for compound **2.**

Supplementary Table 1

Surface-active small molecules in the intestinal tract of animals

Previously-described surface-active gut metabolites from different animals.

	animal	chemical class	reference
**vertebrates**	reptiles and mammals	bile acids and alcohols	2
**invertebrates**	crickets, spiders, crabs	N-acylamino acids	3
	polychaetes	acylamino acids	4
	crabs	acylsarcosyltaurines	5
	snails	alkylsulfuric acid ester type	6
	sea cucumber	alkylsulfuric acid	7
	hepatopancreas of crayfish	furan fatty acids	8

Supplementary Table 2.
Co-localized dialkylfuransulfonic acid metabolites detected by on-tissue desorption electrospray ionization mass spectrometry.

	exact mass measured	expected m/z	mass deviation	sum formula	fragmentation (on tissue (DESI-MS) and in extract (NSI)) *	
					MS^2	MS^3
**1**	259.1010	259.1013	1.3ppm	$C_{12}H_{20}O_4S$	173(Δ98)<br>188(Δ71)<br>244(Δ15)	109 (Δ64), 93 (Δ80), 73 (Δ100)<br>173 (Δ15), 159 (Δ29)<br>173 (Δ71)
**2**	245.0853	245.0854	0.4ppm	$C_{11}H_{18}O_4S$	153(Δ92)<br>173(Δ72)<br>174(Δ71)<br>188(Δ57)<br>201(Δ44)<br>230(Δ15)<br>167(Δ92)	71<br>109<br>110<br>173 (Δ15)<br>n.d.<br>173<br>71 (Δ96)
**3**	275.0959	275.0964	1.9ppm	$C_{12}H_{20}O_5S$	257<br>243<br>231<br>211<br>204<br>190<br>175<br>155	193, 185<br>225, 198, 179<br>148, 80<br>169, 155<br>190, 189, 175, 80<br>175, 160, 147<br>160, 147, 111, 80<br>127, 98
**4**	273.0802	273.0808	2.1ppm	$C_{12}H_{18}O_5S$	258<br>244<br>230<br>209<br>202<br>193<br>188<br>173<br>159<br>129	215, 173, 184<br>188, 173<br>202, 187, 173, 80<br>198, 165, 110<br>173, 159, 187, 80<br>151, 136, 122, 95, 81<br>173<br>158, 145, 109, 93, 80<br>95,81<br>80
**5**	291.0908	291.0912	1.4ppm	$C_{12}H_{18}O_6S$	273<br>249<br>211<br>205<br>183<br>179<br>165<br>155<br>151	245, 209, 198<br>206, 162, 180, 140, 80<br>193, 169, 155<br>141, 111, 80<br>165, 155<br>151<br>147, 137, 123, 109<br>127, 110, 98<br>121, 87, 73, 65
**6**	289.0751	289.0757	2.0ppm	C12H18O6S	271<br>261<br>259<br>257<br>243<br>217<br>202<br>189	255, 243, 227, 151<br>243, 217, 181, 137, 80<br>245, 215, 195, 188<br>239, 213, 193, 186, 137<br>215, 202, 135<br>189, 137<br>187, 173<br>125, 160, 109

*neutral losses observed for compound **1, 2**: (Δ15) - CH_3; (Δ29) - C_2H_5; (Δ71) - C_5H_{11}, (Δ92) – CO_3S

Supplementary references

1. Liebeke, M. & Bundy, J. G. Tissue disruption and extraction methods for metabolic profiling of an invertebrate sentinel species. *Metabolomics* **8**, 819-830 (2012).

2. Hagey, L. R., Vidal, N., Hofmann, A. F. & Krasowski, M. D. Evolutionary diversity of bile salts in reptiles and mammals, including analysis of ancient human and extinct giant ground sloth coprolites. *BMC Evol Biol* **10**, 133 (2010).

3. Collatz, K. G. & Mommsen, T. Structure of emulsifying substances in several invertebrates. *J Comp Physiol* **94**, 339-352 (1974).

4. Smoot, J. C., Mayer, L., Bock, M. J., Wood, P. & Findlay, R. H. Structures and concentrations of surfactants in gut fluid of the marine polychaete *Arenicola marina*. *Marine Ecology-Progress Series* **258**, 161 (2003).

5. van den Oord, A., Danielsson, H. & Ryhage, R. On the structure of the emulsifiers in gastric juice from the crab *Cancer pagurus* L. *J Biol Chem* **240**, 2242-2247 (1965).

6. Collatz, K. G., Mommsen, G. & Mommsen, T. Emulgatoren vom Alkylschwefelsäure- und Glyceridtyp im Darmsaft der Weinbergschnecke *Helix pomatia* L. *J Comp Physiol* **96**, 123-129 (1975).

7. Vonk, H. J. The properties of some emulsifiers in the digestive fluids of invertebrates. *Comp Biochem Physiol* **29**, 361-371 (1969).
